# Centrosomes: Til O-GlcNAc Do Us Apart

**DOI:** 10.3389/fendo.2020.621888

**Published:** 2021-02-01

**Authors:** Aiyun Yuan, Xiangyan Tang, Jing Li

**Affiliations:** Beijing Key Laboratory of DNA Damage Response and College of Life Sciences, Capital Normal University, Beijing, China

**Keywords:** O-linked N-acetylglucosamine, O-linked N-acetylglucosamine transferase, cell cycle, centrosome, cilia

## Abstract

The centrosome apparatus is vital for spindle assembly and chromosome segregation during mitotic divisions. Its replication, disjunction and separation have to be fine-tuned in space and time. A multitude of post-translational modifications (PTMs) have been implicated in centrosome modulation, including phosphorylation, ubiquitination and acetylation. Among them is the emerging O-linked N-acetylglucosamine (O-GlcNAc) modification. This quintessential PTM has a sole writer, O-GlcNAc transferase (OGT), and the only eraser, O-GlcNAcase (OGA). O-GlcNAc couples glucose metabolism with signal transduction and forms a yin-yang relationship with phosphorylation. Evidence from proteomic studies as well as single protein investigations has pinpointed a role of O-GlcNAc in centrosome number and separation, centriole number and distribution, as well as the cilia machinery emanating from the centrosomes. Herein we review our current understanding of the sweet modification embedded in centrosome dynamics and speculate that more molecular details will be unveiled in the future.

## Introduction

The centrosome apparatus, as its name suggests, has been at the center of cell biology (1, 2). Structurally, each centrosome contains two centrioles. In quiescence animal cells, centrioles will form cilia, which function as sensing antenna and have been associated with many ciliopathies (35 types) due to developmental defects or homeostasis imbalance (3). In dividing cells, centrioles build the centrosomal structure by assembling the pericentriolar material (PCM) matrix around it, thus comprising the microtubule organization centers and exerting various cellular effects *via* the microtubule cytoskeleton system. Disruption of the centrioles or centrosome structures will wreak havoc on many biological processes (1, 2). The centrosome cycle has to be in perfect synchrony with the cell cycle (4). It replicates as DNA synthesizes, and both occur once and only once per cell cycle. It segregates as chromosomes separate, and emanates the spindle apparatus to ensure faithful segregation of the sister chromatids. Therefore, the number and the timing of separation have to be under stringent control so that a successful mitosis will ensue.

Previous investigations have unveiled that duplication, maturation, disjunction, separation and degradation of centrosomes are fine-tuned by an intricate network of kinases, phosphatases, ubiquitin E3 ligases (5) as well as O-linked N-acetylglucosamine (O-GlcNAc) transferase (OGT). OGT is the only writer for the O-GlcNAc modification. About 2–5% of the glucose we consume each day is shunted into the hexosamine biosynthetic pathway (HBP) to generate UDP-GlcNAc (6, 7), which is the donor group of the GlcNAc moiety to the Ser/Thr residues of substrate proteins. Now four decades’ foray into this mysterious modification has unveiled that it could crosstalk with other post-translational modifications (PTMs), and regulate various biological processes, such as transcription, cell cycle and stress response. Not surprisingly, it underlies cancer, neurodegenerative diseases and diabetes (6, 7). However, how O-GlcNAc dictates various aspects of centrosome dynamics has been anecdotal. Here we review the sweet bits and pieces of centrosome biology and attempt to fathom our future strides forward.

## O-GlcNAc Mediates the Centrosome Number

The first inkling of implication of OGT in the centrosome comes from cytology, as it was found in 2005 that OGT localizes to the centrosome (8) and later to the mitotic spindle (8, 9). What is perplexing is that both OGT and OGA overproduction cause supernumerary centrosomes (10), suggesting that it is not a linear relationship between O-GlcNAc levels and centrosome numbers. It is a possible scenario that some centrosome assembly proteins are under dynamic regulation of both OGT and OGA.

Then in 2010, a large scale proteomic study aiming to elucidate the mitotic role of OGT was carried out (9), and 141 new proteins involved in spindle and cytokinesis were pinpointed to be regulated by O-GlcNAcylation. Of particular interest is the nuclear mitotic apparatus protein (NuMA1). NuMA1 is O-GlcNAcylated at Ser1844, as revealed by the glycoproteomic studies (9), and later shown to regulate spindle pole cohesion (11). O-GlcNAcyated NuMA1 associates with Galectin-3 that belongs to the lectin family. In the O-GlcNAc-defective S1844A mutant of NuMA1, the partnership between NuMA1 and Galectin-3 is abolished and the spindle pole cohesion is defective, resulting in multipolar spindles. These results suggest that centrosomal Galectin is pivotal for centrosome integrity, *via* O-GlcNAcylated NuMA1 (11).

Besides centrosomal components, kinases also fine-tune the centrosome structures. Aurora B, a member of the chromosomal passenger complex (CPC) that coordinates the intricate process of spindle assembly and disassembly (12), complexes with both OGT and OGA (13). Furthermore, the mitotic master kinase—polo-like kinase 1 (PLK1) (5) is O-GlcNAcylated *in vitro* (14). But whether the interaction between these kinases and OGT directly regulates centrosome dynamics still warrants further investigations.

## O-GlcNAc Governs Centrosomal Distances

Totally unexpected, research from our lab discovered Thiamet-G (OGA inhibitor) treatment will significantly distance the two centrosomes during interphase (15). This untimely event will have deleterious effects on the subsequent chromosome segregation. As the segregation of centrosomes are dictated by PLK1 (5), we reasoned the premature centrosome separation phenotype could be due to an upregulation of PLK1 kinase activity. Since PLK1 kinase activity is regulated by phosphorylation at T210 in its kinase domain (16), and T210 is dephosphorylated by the Myosin Phosphatase Targeting Subunit 1 (MYPT1)-Protein Phosphatase 1 cβ (PP1cβ) complex (17), we investigated MYPT1 O-GlcNAcylation.

Although O-GlcNAcylation of MYPT1 was reported in 2008 (18), its modification site and function are yet to be revealed. In perfect timing with our investigation, chemical biology approaches utilizing metabolic glycan labeling and chemoenzymatic labeling together with click chemistry has witnessed a surge of O-GlcNAc profiling work (19, 20), and among the proteins idenfied is MYPT1. By trial-and-error mutagenesis studies, we showcased that MYPT1 is O-GlcNAcylated at four major sites: T577, S585, S589 and S601. The perfect marriage between chemistry and biology will certainly invite many fresh opportunities.

**Figure 1:**
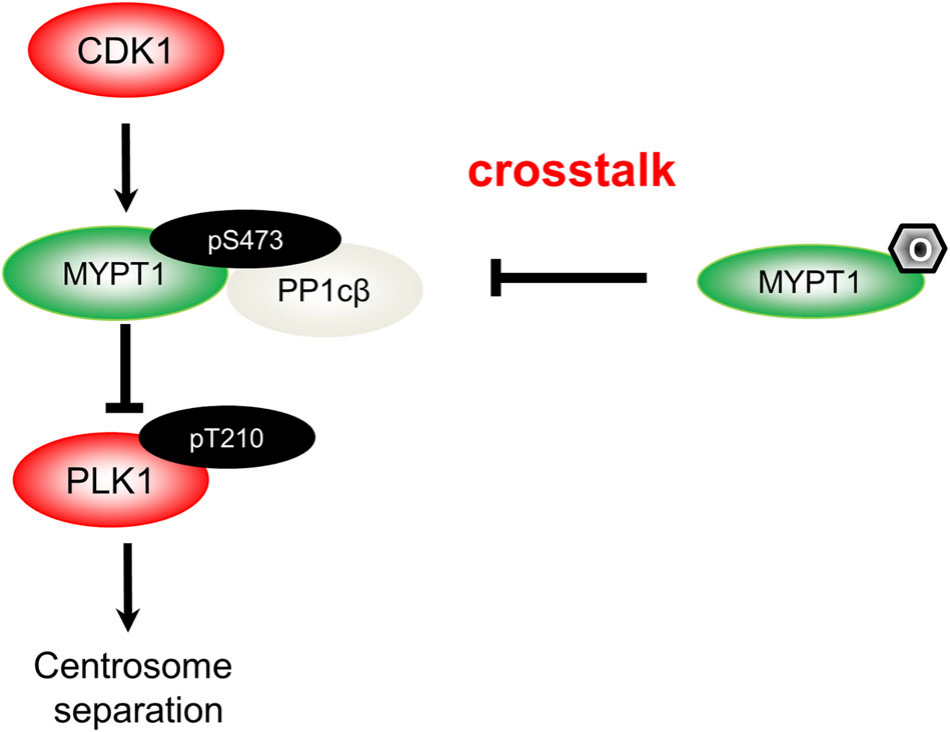
A diagram spotlights the role of O-GlcNAcylation in centrosome separation based on our work (15). During mitosis, CDK1 phosphorylates MYPT1 at pS473 to be docked onto PLK1. Hence, MYPT1 recruits PP1cβ to dephosphorylate and inactivate PLK1 (17). MYPT1 has been shown to be O-GlcNAcylated (18). In our recent work (15), we demonstrated that MYPT1 O-GlcNAcylation at four major sites antagonizes pS473, dissociates MYPT1 from PLK1. Thus by elevating PLK1 pT210, O-GlcNAcylation induces untimely separation of centrosomes.

The mechanism of MYPT1- PP1cβ to dephosphorylate PLK1 has been unravelled: cyclin-dependent kinase 1 (CDK1) will phosphorylate MYPT1 at S473, thus creating a binding pocket, and subsequently docks MYPT1 to the C-terminal polo-binding domain (PBD) of PLK1 (17). MYPT1 then recruits PP1cβ, bridging the phosphatase and its substrate, and PLK1 will then be dephosphorylated (17). By biochemical assays, we provide incisive evidence that O-GlcNAcylation almost totally negates phosphorylation effects. By antagonizing pS473, O-GlcNAcylation disjoins MYPT1 from PLK1, thus maintaining PLK1 pT210 activity (15) (**Figure 1**).

Almost concomitantly, Pedowitz et al. (21) also studied MYPT1 O-GlcNAcylation. They unraveled that under basal conditions it antagonizes pT696, which is an inhibitory phosphorylation for the myosin phosphatase, and controls actin contraction. Like two sides of the same coin, these two stories reveal that one modification could carry out disparate physiological functions during distinct biological processes.

## O-GlcNAc Negatively Regulates Cilia in Quiescent Cells

Two papers came out in 2018 and 2019, describing that O-GlcNAc exerts a negative effect on ciliogenesis and cilia lengths (22, 23). Yu et al. (22) first utilized DB mice that harbor diabetic mutations in the leptin receptor, and noticed that DB mice have fewer and shorter cilia than wild type. They further exploited human retinal pigment epithelial (RPE-1) cells, and serum-starved them in conjunction with distinct glucose concentrations. Again, high glucose induces fewer and shorter cilia. In the follow-up investigations, they manipulated cellular O-GlcNAc levels *via* different approaches—glucosamine (a precursor of UDP-GlcNAc), siRNA targeting *OGA*, TMG and BZX (OGT inhibitor). And every time, they observed the consistent phenotype. Cytologically, OGT is also discernable at the basal body where cilia emanate. Lastly, by feeding the DB mice with BZX, Yu et al. discovered that decreasing O-GlcNAc levels could rescue the ciliary defects partially. Thus the convergence of diabetes and ciliopathy phenotypes might lie upon O-GlcNAc imbalance.

Tian et al. took a different angle of attack. They adopted hTERT-RPE1 and IMCD3 cells, and used siRNA targeting *OGT*, OGA inhibitors including TMG and GlcNAcstatin G (SG), and alloxan (used in this study as an OGT inhibitor) to measure the ciliary effect of O-GlcNAcylation. Although we need to take the alloxan result with a bit of caution (Alloxan is not a specific OGT inhibitor), Tian et al. also discovered a negative effect on cilia by O-GlcNAc (23), congruent with the results from Yu et al. (22). Mechanistically, α-tubulin and histone deacetylase 6 (HDAC6) are found to be O-GlcNAcylated. As HDAC6 is the main deacetylase for axonemal microtubules, they demonstrated that HDAC6 O-GlcNAcylation promotes its deacetylase activity and thus microtubule disassembly, culminating in cilia shortening (23). Currently, the O-GlcNAcylation sites of α-tubulin and HDAC6 remain to be explored.

## O-GlcNAc Regulates Centriole Numbers and Distribution

In a follow-up investigation by Yu et al. (24), *Ogt^+/-^* mice were generated. Again, shorter ciliary lengths and fewer ciliary numbers were observed. When they used mouse tracheal epithelial cells (MTECs) as a model to study cilia biogenesis, upon OGT inhibition by BZX or OSMI-1, the number and distribution of centrioles were aberrant (24). Intriguingly, scanning electron microscopy (SEM) reveals that BZX induces abnormal bulbs at the cilia tips, suggestive of defective intraflagellar transport (IFT), the intricate process to move particles from the cellular body to and fro at the cilia or flagellum tip (25). An in-depth characterization of OGT centrosomal localization was carried out with a three-dimensional structured illumination microscopy (3D-SIM) in G0/G1 phase of U2OS cells, and OGT localizes to the outskirts of the PCM, forming a unique and distinct localization pattern with the known centriole components (25). Although both elegant cytological and thorough animal studies caught OGT at the scene of centrosomes in this paper, a biochemical mechanistic view is still lacking, probably due to the scanty protein amounts in the centriolar structures.

Based on the current data available, it is conceivable that O-GlcNAcylation has a negative impact on cilia, as the cilia is the organelle that reaches out for signals when cells starve. Intuitively, O-GlcNAcylation levels, as a nutrient rheostat, signal the nutrient levels to the cells, so the cilia could be shorter in times of abundance. The secretive role of OGT in cilia might be just unfolding, as many a kinase that localizes to the cilium or regulates ciliogenesis have been shown to be O-GlcNAcylated, including protein kinase B (PKB/AKT) (26) and hypoxia-inducible factor α (27). We reckon these kinases might also be affecting downstream pathways to adjust the metabolic flux in accord with O-GlcNAcylation levels. Alternatively, as Yu et al. (24) suggests, OGT substrates might lie in the IFT pathway. A screen might be needed to identify which IFT proteins are subject to O-GlcNAcylation.

## Conclusions

**Figure 2:**
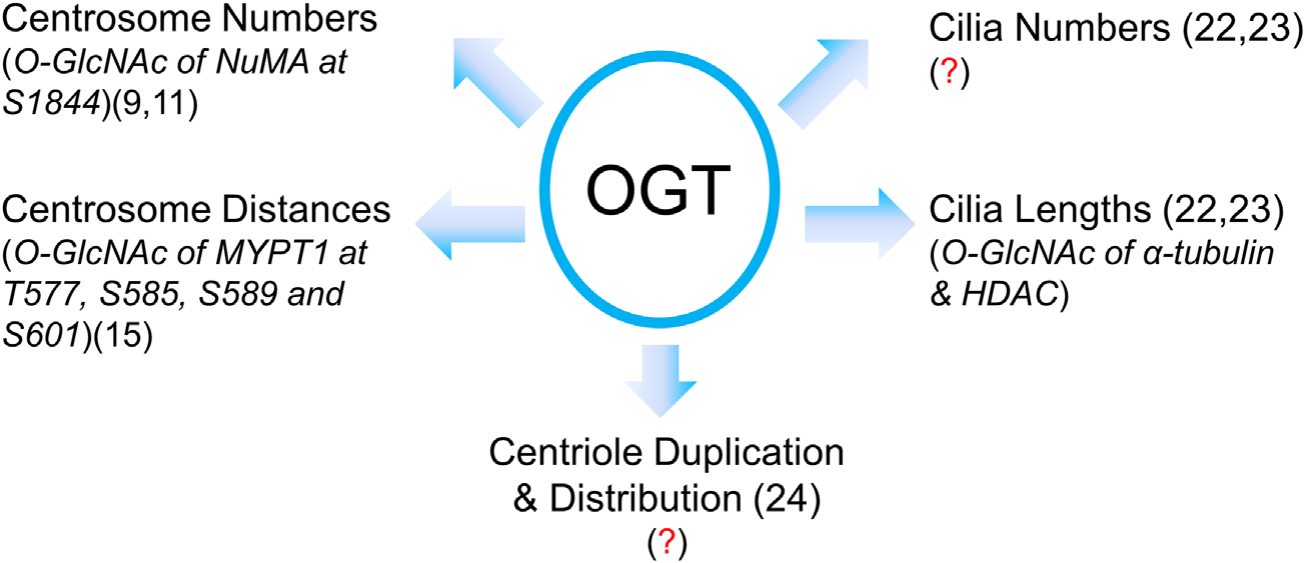
A summary of our current understanding of OGT in centrosome biology in its infancy. Present literature suggests that OGT regulates centrosome numbers and distances, cilia numbers and cilia lengths, centriole numbers and distribution. However, due to the limited amount of proteins in the centrosome or cilia, identification of OGT substrates in these vestigial subcellular structures has been quite a daunting task. The known O-GlcNAcylated proteins with their modification sites are italicized with the proper references. Red question marks demarcate where mechanistic insights are still lacking.

The role of OGT in the centrosome is far from complete (**Figure 2**). Our review is intended as a beginning for this area of research in its infancy, rather than an end or even a mid-point. With so many questions crying to be answered, we need more intricate tools to manipulate the fine and subtle structures of centrosomes or cilia. We speculate that OGT substrates may encompass dynamic IFT components or kinases that mediate cilia biogenesis, and we will not be surprised if OGT catalyzes some the core proteins in the centrosome proteome (28).

Indeed, the convergence of chemistry and biology is ushering in a new era for our study. As chemical biology has enabled O-GlcNAc profiling within reach (19, 20), new chemical biology tools might circumvent questions that have impeded biologists, such as low protein amounts or low O-GlcNAc stoichiometry. As the entanglement of O-GlcNAc with other PTMs is ever increasing, we expect more molecular details will be revealed in the future to form a conceptual framework to understand this entrancing question.

## Author Contributions

AY, XT, and JL wrote the manuscript together. All authors contributed to the article and approved the submitted version.

## Funding

This work was supported by the National Natural Science Foundation of China (NSFC) fund (31872720).

## Conflict of Interest

The authors declare that the research was conducted in the absence of any commercial or financial relationships that could be construed as a potential conflict of interest.

## Abbreviations

O-GlcNAc, O-linked N-acetylglucosamine; OGT, O-GlcNAc transferase; OGA, O-GlcNAcase; PBD, polo-binding domain; TMG, Thiamet-G; CDK1, cyclin-dependent kinase 1; MYPT1, Myosin Phosphatase Targeting Subunit 1; PP1cβ, Protein Phosphatase 1 cβ; PTM, Post-translational modification.
